# Ferroptosis blockade: beyond preservation to active graft therapy

**DOI:** 10.1038/s41392-026-02996-8

**Published:** 2026-09-14

**Authors:** Chen Xing, Gabriel C. Oniscu, Volker M. Lauschke

**Affiliations:** 1 Dr. Margarete Fischer-Bosch Institute of Clinical Pharmacology, Stuttgart, Germany; 2 Department of Physiology and Pharmacology and Center for Molecular Medicine, Karolinska Institutet and University Hospital, Stockholm, Sweden; 3 University of Tübingen, Tübingen, Germany; 4 Division of Transplantation Surgery, Department of Clinical Science, Intervention and Technology, Karolinska Institutet, Stockholm, Sweden; 5 Department of Transplantation Surgery, Karolinska University Hospital, Stockholm, Sweden; 6 Department of Pharmacy, the Second Xiangya Hospital, Central South University, Changsha, China

**Keywords:** Drug development, Translational research

In a recent study published in Cell, Veeckmans et al. show that small molecule ferroptosis inhibitors can improve liver and lung graft function.^1^ These results demonstrate that ferroptosis is an emerging targetable driver of ischemia–reperfusion injury (IRI) and highlight the critical role of ex vivo organ perfusion as a therapeutic window for graft repair before transplantation.

Organ transplantation is limited by organ availability, compounded by significant changes in the demographics of the donor population. Marginal organs, prolonged preservation, donation after circulatory death and IRI all increase the risk of early allograft dysfunction in these sensitive donor organs. Machine perfusion has revolutionized the field by enabling a dynamic assessment and potential resuscitation of the donor organs. Yet, most perfusion strategies are only supportive by providing oxygenation and, in the case of normothermic perfusion, some nutrients, and by monitoring of the organs to be transplanted. There is, however, a huge unrealized potential to intervene and tackle the mechanisms of IRI and potentially improve graft performance beyond what is currently achieved only with dynamic perfusion. In their recent study in *Cell*, Veeckmans et al. identify ferroptosis as a therapeutically actionable mechanism of graft injury and demonstrate that pharmacological inhibition of ferroptosis can improve graft performance by focusing on liver and lung.

Ferroptosis is an iron-dependent form of regulated cell death due to phospholipid peroxidation and the resulting accumulation of lipid peroxides in cellular membranes.^2^ In contrast to apoptosis and necroptosis, ferroptosis is not driven by caspases or the necrosome. Instead, ferroptosis is facilitated by an enrichment of intracellular redox-active iron and an accumulation of polyunsaturated fatty acids in membrane phospholipids in the background of deficient antioxidant capacity. Combined, these factors result in compromised lipid redox homeostasis and the formation of lipid radicals, which amplify membrane damage. This is particularly relevant to transplantation where transient ischemia perturbs energy metabolism, antioxidant capacity and mitochondrial function, while subsequent reperfusion abruptly restores oxygenation, resulting in oxidative injury. The resulting lipid peroxidation can damage parenchymal, endothelial and stromal compartments, which compromises the microvascular integrity and the function of the transplanted organ.

**Fig. 1:**
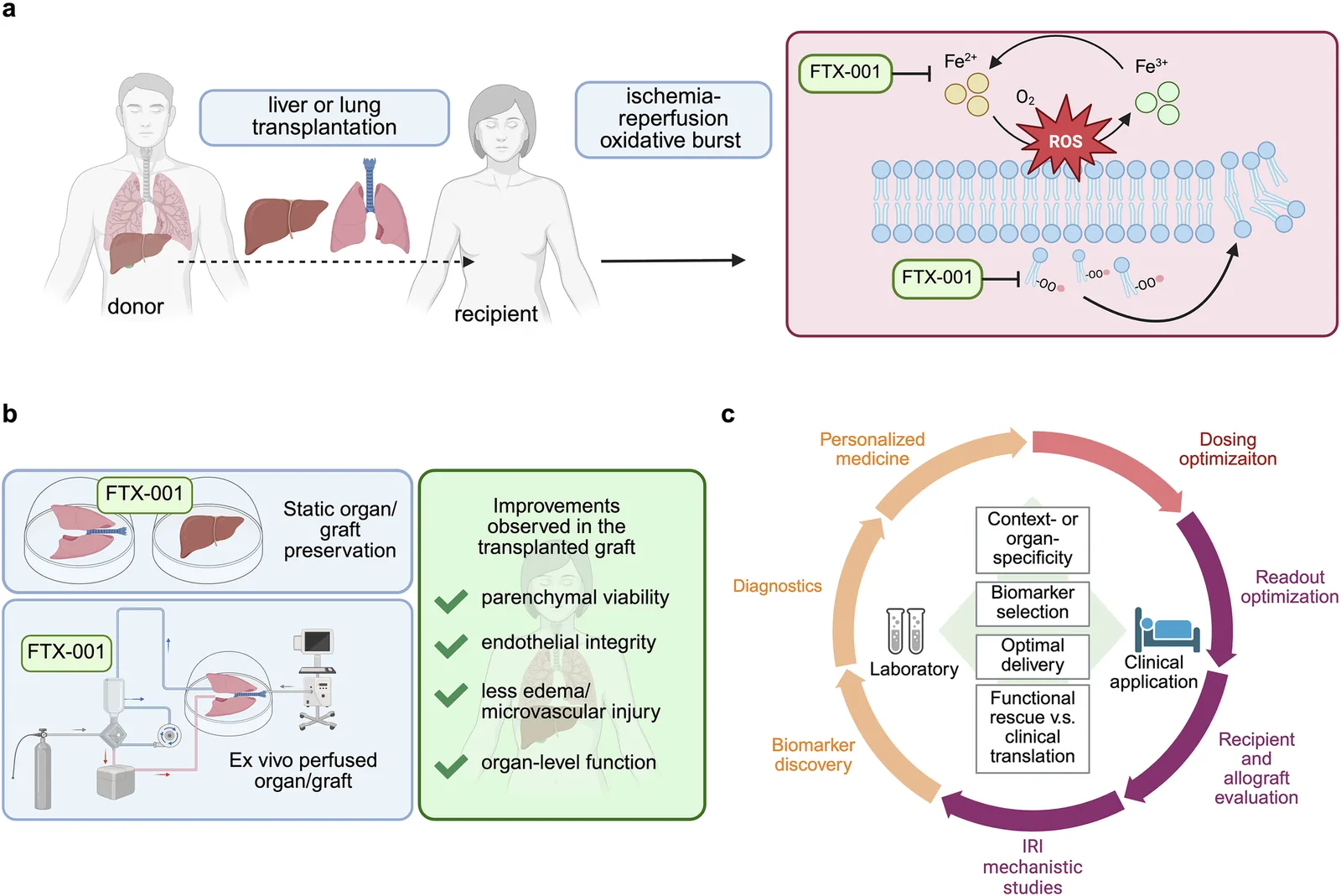
Ferroptosis blockade as an ex vivo graft-conditioning strategy for liver and lung transplantation. **a** During liver or lung transplantation, ischemia–reperfusion injury (IRI) disrupts energy and redox homeostasis and promotes reperfusion-associated oxidative stress. Redox-active iron, oxygen-derived ROS, and oxidizable phospholipids converge to drive lipid radical propagation and lipid peroxide accumulation in cellular membranes, resulting in ferroptotic graft injury. FXT-001, a ferrostatin-1 analog with dual radical-trapping and iron-binding activity, interrupts this cascade by limiting iron-dependent radical generation and membrane lipid radical propagation. **b** FXT-001 is illustrated as an ex vivo graft-conditioning intervention evaluated in static preservation and perfusion-based liver and lung graft models. By suppressing ferroptosis-associated lipid peroxidation, FXT-001 attenuates graft injury and preserves viability- and function-associated readouts, including parenchymal viability, endothelial and microvascular integrity, reduced edema-associated injury, and organ-level performance. **c** Further translation will require the elucidation of the donor organ- and context-dependency of ferroptosis, the selection of robust pharmacodynamic biomarkers for graft stratification and dosing, titration of optimal timing and the determination of how to translate improved graft function into histological improvement and clinical benefit after transplantation. The figure was generated by BioRender. The figure can be viewed at Xing, C. (2026) https://BioRender.com/m7aw1yr

Veeckmans et al. provide several lines of evidence that ferroptosis not only marks injured tissue, but that it is an early and targetable event (Fig. 1a). In human liver transplantation, they detect a rapid and transient increase in lipid peroxidation during the early reperfusion phase. These kinetics suggest that inhibition of ferroptotic injury within this clinically accessible window might provide opportunities to reduce the likelihood of irreversible graft failure. The authors further validate ferroptosis-associated injury across experimental models, supporting the idea that lipid peroxidation is a conserved component of graft IRI rather than a liver-specific phenomenon.

FXT-001, a best-in-class analog of ferrostatin-1 with dual radical-trapping and iron-binding properties has been previously demonstrated to inhibit ferroptosis and reduce IRI in animal models.^3,4^ Iron-dependent radical generation promotes lipid peroxidation, which in turn drives ferroptosis. Thus, a compound that can target both of these mechanistically linked events separately may be more effective than generic antioxidants or agents interfering with only one of these processes. In clinically relevant large-animal models, FXT-001 attenuated liver injury and improved functional parameters during ex situ reperfusion mimicking transplantation. In lung graft models, the compound similarly reduced features associated with organ injury, including parameters linked to edema and tissue function. These results suggest that inhibition of ferroptosis can promote physiological maintenance of the to-be-transplanted organ (Fig. 1b).

A particularly compelling aspect of the work is its use of ex situ perfusion of human donor lungs that were declined for transplantation. Split-organ or paired-organ perfusion designs are powerful models because they reduce inter-donor variability and allow direct comparison of treated and untreated tissue under identical conditions. In this setting, FXT-001 preserved viability-associated features relative to untreated controls. Although such experiments do not replace prospective transplantation studies, they provide an important bridge between animal models and clinical translation. They also illustrate why transplantation and especially ex situ organ perfusion models are an unusually attractive setting for ferroptosis-targeted therapy, as the drug can be delivered directly to the organ during machine perfusion, before implantation, maximizing the local benefit while limiting the systemic exposure in the recipient.

The study also advances the medicinal chemistry of ferroptosis inhibition. Many experimental ferroptosis inhibitors have been useful as biological tools but have unsuitable drug disposition profiles or unclear safety profiles. Veeckmans et al. report next-generation analogs, FXT-002 and FXT-003, with improved pharmacokinetic and safety characteristics. This is important because clinical translation will require not only pathway validation but also compounds compatible with regulatory development, scalable production and defined exposure–response relationships. The ex vivo perfusion setting may reduce some barriers, but dose, timing, metabolism, tissue penetration and washout will still require careful optimization.

Several implications follow from this work. First, organ preservation should increasingly be viewed as an active therapeutic phase rather than a passive interval between organ procurement and implantation. The rapid expansion of ex situ machine perfusion creates an optimal platform for controlled, targeted intervention, pharmacodynamic monitoring and functional readout. Second, ferroptosis inhibition could help expand the donor pool by rescuing organs currently considered too vulnerable to IRI and at high risk of failure. Third, ex situ organ perfusion may become a human translational model for ferroptosis-targeted therapy in broader ischemic diseases, including myocardial infarction, stroke, vascular surgery and shock-associated organ injury.

Important questions remain. Mechanistically, ferroptosis intersects with mitochondrial dysfunction, complement activation, endothelial injury, innate immune signaling and other regulated cell-death pathways. The relative contribution of ferroptosis will likely differ by organ, donor type, ischemic duration, perfusion temperature and baseline tissue quality, incentivizing investigations into organ-specific benefits of ferroptosis inhibitors. Secondly, lipid peroxidation is central to ferroptosis but is not always specific for ferroptotic cell death. Robust pharmacodynamic markers will be needed to guide dosing and to identify the grafts most likely to benefit. Thirdly, in this study, the ferroptosis inhibitor was administered during the static preservation of the graft and not during machine perfusion. Further experimental work is needed to define the optimal time of administration in the donor, during graft preservation, during machine perfusion or in the recipient and if sequential doses lead to an improved effect as compared to the “hit and run” scenario in this study. Finally, the observed improvement in graft function failed to translate into improvements in histopathology which may suggest that further refinements of this potential strategy are needed and clinical studies must determine whether improved ex vivo function translates into reduced early allograft dysfunction, better graft survival and improved recipient outcomes (Fig. 1c).

Nevertheless, Veeckmans et al. provide a strong conceptual and translational advance. By connecting early lipid peroxidation in human transplantation with druggable ferroptotic injury, and by demonstrating organ protection in liver and lung perfusion systems, the study establishes ferroptosis blockade as a promising graft-conditioning strategy. More broadly, it exemplifies a new direction in transplantation medicine: using machine perfusion technology not only to preserve organs, but to pharmacologically repair them before transplantation.
